# iCTNet2: integrating heterogeneous biological interactions to understand complex traits

**DOI:** 10.12688/f1000research.6836.2

**Published:** 2015-09-28

**Authors:** Lili Wang, Daniel S. Himmelstein, Adam Santaniello, Mousavi Parvin, Sergio E. Baranzini

**Affiliations:** 1 School of Computing, Queen’s University, Kingston, Ontario, K7L 3N6, Canada; 2 Department of Neurology, University of California San Francisco, San Francisco, CA, 94158, USA; 3 Graduate Program in Biological and Medical Informatics, University of California, San Francisco, San Francisco, CA, 94143-0523, USA

**Keywords:** big data integration, heterogeneous network, drug re-purposing, disease ontology

## Abstract

iCTNet (integrated Complex Traits Networks) version 2 is a Cytoscape app and database that allows researchers to build heterogeneous networks by integrating a variety of biological interactions, thus offering a systems-level view of human complex traits. iCTNet2 is built from a variety of large-scale biological datasets, collected from public repositories to facilitate the building, visualization and analysis of heterogeneous biological networks in a comprehensive fashion via the Cytoscape platform. iCTNet2 is freely available at the Cytoscape app store.

## Introduction

In the past decade, an exponential increase in the amount and variety of publicly available genomic, transcriptomic, proteomic and other ‘omics’ data has occurred, altogether encompassing a wide range of biological interactions. Each dataset captures distinct features of molecular functions involved in complex traits, with the goal of describing and ultimately understanding biological complexity. However, these datasets are mostly used in isolation, and even the integration of any two of them would take a significant effort for the average biological investigator.

Previous work in this area is largely limited to merging data of only two types. Goh *et al.* ^1^ built the first “Diseasome”, a bipartite network of diseases and their associated genes. Lage *et al.* ^2^ merged protein-protein interaction networks with disease-gene associations. Similar approaches have been taken to integrate genes (transcripts) with tissue ^3^ and miRNA ^4^. More recently, drug-target (and drug-side effects) networks have attracted attention due to the potential of this approach to illuminate on candidates for drug repositioning ^5, 6^. While the integration of heterogeneous biological interactions would be key in fueling practical applications of systems biology from rational drug discovery to disease risk prediction, dedicated approaches and tools to accomplish this task are only starting to emerge.

Heterogeneous data sets can be joined based on common keys (i.e., identifiers or ontology terms), but the integration of large-scale biological interactions is time-consuming, and particularly hampered by the lack of universal identifiers in different repositories. We previously described the integrated Complex Trait Networks (iCTNet) as an attempt to capture multiple biological relationships available in the public domain. In the original version of iCTNet ^7^, five types of biological interactions (protein-protein, disease-gene, drug-gene, tissue-gene, disease-tissue) were integrated in a graph fashion, allowing for practical and intuitive integration of those data sources within the Cytoscape 2 environment. We argue that incorporating an expanded roster of popular databases would maximize the utility of this tool in many ways. Such integration of heterogeneous interactions would further accelerate our understanding of complex traits, and ultimately enable development of predictive disease models and facilitate drug discovery and repositioning. In this study, we present iCTNet2, a Cytoscape 3 app and database incorporating nine different types of interactions among six different types of entities: phenotypes, genes (proteins), miRNAs, tissues, drugs, and drug side effects. In addition to increasing the size of the database by a factor of 10, a central and distinctive feature of iCTNet2 is the incorporation of disease and anatomical ontologies as scaffolds onto which the different data types are integrated.

## Material and methods

### Overview of iCTNet2

**Figure 1.**
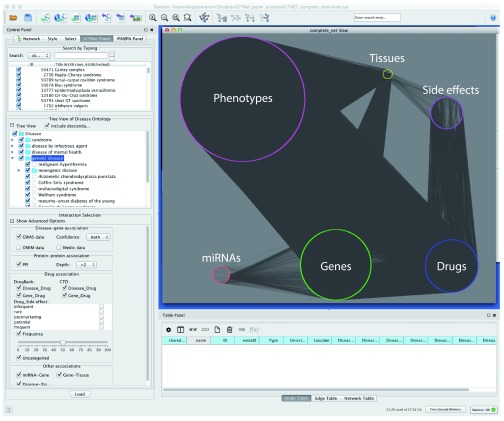
iCTNet 2.0 screenshot.

iCTNet2 app is an update to the iCTNet plugin for Cytoscape2. The app was developed in Java version 7 for Cytoscape 3. The core of iCTNet2 is the iCTNet2 database, which can be accessed via the iCTNet2 app from Cytoscape ^8^, through a user-friendly graphical interface ( Figure 1). iCTNet2 app uses the Model-view-controller (MVC) pattern, dividing the app into three parts. The Model objects represent the data structures of a variety of biological entities and interactions. The View objects include three panels, where the user can search and select entities. The Control objects inherit org.cytoscape.work.AbstractTask class, implementing the database connection and the communication between the Model and the View.

### Database

All data resources have been processed and stored in a relational MySQL ( http://www.mysql.com) database system. Currently, the iCTNet2 app is the only available access to the iCTNet2 database. The database schema has been designed using MySQL Workbench 5.2 ( http://www.mysql.com/products/workbench). All the queries are executed in terms of stored procedures through JDBC API. Once the user clicks the “Load” button, the data is queried and loaded into Cytoscape. The iCTNet2 database collects a variety of large-scale biological datasets from public repositories to facilitate the building, visualization and analysis of heterogeneous biological networks. Additionally, iCTNet2 incorporates the disease ontology (DO) ^9^ as the primary vocabulary for cataloguing phenotypes in a tree-like structure. Table 1 lists the publicly available resources used to build the iCTNet2 database.

**Table 1.** The data resources collected in iCTNet2.

	Type	Resources	Version/Date	URL
nodes	Phenotype	Disease Ontology	2013-12-12	http://disease-ontology.org/
	Gene	HGNC(including non-coding)	2014-02-05	http://www.genenames.org/
	miRNA	mirCat	2013-11-11	http://www.mirrna.org
	Tissue	BRENDA Tissue Ontology	2013-10-09	http://www.brenda-enzymes.org
	Drug	CTD	2013-12-20	http://ctdbase.org/
	Side effect	Medical Dictionary for Regulatory Activities(MedDRA)	MedDRA 16.1	http://www.meddra.org
	Side effect	UMLS Metathesaurus	2011AB	http://www.nlm.nih.gov/research/umls/
edges	Phenotype-gene	GWAS Catalog	v1.0.1: 2015-07-08	http://www.genome.gov/gwastudies/
	Phenotype-gene	OMIM	2013-11-11	http://www.omim.org/
	Phenotype-gene	CTD	2013-12-20	http://ctdbase.org/
	Phenotype-tissue	Ontology Inference	
	Gene-tissue	GNF Gene Atlas	2010-02-01	http://www.gnf.org/
	Drug-phenotype	CTD	2013-12-20	http://ctdbase.org/
	Drug-gene	CTD	2013-12-20	http://ctdbase.org/
	Drug-gene	DrugBank	2012-08-10	http://www.drugbank.ca/
	Drug-side effect	SIDER	SIDER 2: 2012-10-17	http://sideeffects.embl.de/
	Side effect-tissue	Ontology Inference	
	Protein-protein	iRefIndex ppiTrim	iRefIndex 12.0	http://irefindex.org http://www.ncbi.nlm.nih.gov/CBBresearch/ Yu/downloads/ppiTrim.html
	miRNA-gene	mirCat		http://www.mirrna.org

### Types of nodes

***Phenotypes/diseases.*** In addition to DO, we also included two other disease vocabularies: the Experimental Factor Ontology (EFO) and MEDIC. EFO is an ontology developed by the European Bioinformatics Institute (EBI) with a detailed disease component ^10^. MEDIC is a list of vocabularies produced by the Comparative Toxicogenomics Database (CTD) ^11^ which incorporates disease terms from the Online Mendelian Inheritance in Man (OMIM) ^12^ and the U.S. National Library of Medicine’s Medical Subject Headings (MeSH) ( http://www.nlm.nih.gov/mesh/). The DO includes OMIM cross-references, thus providing the mapping for our network. DO cross-references were mapped onto OMIM and MeSH to provide mappings to MEDIC. Since the DO did not include direct mappings to the EFO, relevant EFO terms were manually mapped. Our mapping currently only covers the subset of EFO disease terms available in the GWAS catalog as of Dec 2014 ^13^ (We submitted these 137 mappings to the DO, which now includes them as cross-references). In total, there are 6,338 phenotype records in the iCTNet2 database.

***Gene.*** Gene names are obtained from the HUGO Gene Nomenclature Committee’s list of human genes (HGNC). iCTNet2 only includes currently valid genes, but also incorporates outdated gene symbols and synonyms into an alias table for reference. Non-protein coding genes are included as well. In order to map symbols or identifiers across different data resources, genes are identified using the integer portion of their HGNC IDs ^14^. The iCTNet database includes 38,079 gene records.

***miRNAs.*** miRNAs and their targets are collected from an online database miRCat ( http://www.mirrna.org), which in turn, assembles data from five databases: microRNA.org, miRTarBase, tarbase, microT (v3.0) and miR2Disease.

***Tissues.*** Tissue types were taken from BRENDA tissue ontology ^15^. We rooted the ontology at ‘whole body’ (BTO:0001489) to exclude the non-animal tissue portions of the ontology.

***Drugs.*** We used the CTD as the primary resource for drugs as references to DrugBank ^16^ identifiers are provided thus facilitating the mapping between these two resources. Therefore, iCTNet2 contains information of 151,378 drugs in total. However, the function of only 10% of them is currently associated (mapped) to genes. Mapped drugs in iCTNet include over 13,000 curated chemicals and associations with several other major chemical databases. While DrugBank 3.0 contains fewer entries than CTD, it has extensive information on most FDA approved therapeutics.

***Side effects.*** The side effect ontology is retrieved from the Medical Dictionary for Regulatory Activities (MedDRA) ( http://www.meddra.org). While providing a high quality and widely adopted vocabulary, the commercial nature of this resource prevents large-scale republication of its terms. Instead, our database reports the Unified Medical Language System (UMLS) ( http://www.nlm.nih.gov/research/umls/) concepts for side effects. Since MedDRA is a source vocabulary for the UMLS, the mapping is straightforward and reversible. Nonetheless, upon request we will provide researchers who have a valid MedDRA license with an untranslated version of our database which includes the hierarchical relationships between side effects.

### Types of interactions

***Phenotype-gene (n=17,778).*** The phenotype-gene associations are the primary resources to study the genetic factors of complex traits. iCTNet2 merges phenotype-gene associations from three online databases: GWAS Catalog, OMIM and CTD. Only CTD relationships with direct evidence of "marker/mechanism" were included. To convert from SNP to gene associations, we combined overlapping loci for each GWAS Catalog disease as recently described ^17^. The author reported gene for each loci was selected as the primary association for each disease.

***Phenotype-tissue (n=5,377).*** These edges represent physiopathological information (i.e. which tissues/organs are likely affected by each disease). To identify tissue relationships with diseases and side effects, we used an ontology inference method. Anatomical disease and side effect terms were manually mapped to their affected tissues in the BTO. For example, connective tissue disease (DOID:65) was mapped to connective tissue (BTO:0000421). Affected tissues were propagated to more specific terms, so only high-level DO and MedDRA terms required manual mapping. See Data S1–Data S3 for the complete mappings.

***Gene-tissue (n=108,400).*** iCTNet2 collects an extensive atlas of tissue-specific gene expression from the GNF gene atlas ^18^. The expression patterns of 79 human tissues are available that can provide important clues about gene functions.

***Drug-disease (n=11,701).*** The drug-disease interactions (indications) are collected from CTD, which in turn, are manually curated from the literature.

***Drug-gene (n=3,426).*** The drug-gene interactions are assembled from CTD and DrugBank, two major databases containing drug information.

***Drugs-side effects (n=1,828).*** The side effects of drugs in humans are an essential source to understand human phenotypes. iCTNet2 collects the information of 888 drugs and 1,450 side effect terms from the side effect resource (SIDER) ^19^, with available side effect frequency.

***Protein-protein interactions (PPI) (n=98,228).*** PPIs are among the most studied interactions in network biology, although the known interactions may present only one tenth of the entire interactions. PPIs are collected from ppiTrim ^20^, which further curates iRefIndex ^21^, a master database consolidating interactions from 15 different sources (including BIND, HPRD, etc).

***miRNA-gene (n=2,457).*** MicroRNAs (miRNAs) are short RNA sequences that regulate the expression of target genes. miRNA-gene interactions are collected from the online database miRCat.

### Database

All data resources have been processed and stored in a relational MySQL ( http://www.mysql.com) database system. Currently, the iCTNet2 app is the only available access to the iCTNet2 database.

### Visualizations

Once installed, iCTNet2 will show up automatically on the left hand side of the Cytoscape window. So through the Cytoscape platform, networks constructed via iCTNet2 can be visualized in different layouts with many visualization features. Cytoscape built-in functions or analysis apps can be easily applied as well.

## Results

iCTNet 2.0 is an updated, expanded and improved version of the Cytoscape 2.x plugin our group developed ^7^. In this new version, developed as a Cytoscape 3.x App, a user can select and download relationships across several biological entities (e.g. diseases, genes, drugs, side effects, etc) to create a heterogeneous network that can be displayed in Cytoscape for further analysis. iCTNet 2.0 can be used to generate new hypothesis about disease relationships, shared pathogenic mechanisms, or prioritize drugs for drug repurposing. In addition, this app can be used to visualize all known information about a particular disease, or process and create publication-ready figures. There are three options to start building networks with iCTNet2. As the metagraph (the graph describing the interactions among the different node types) can be cyclic ( Figure 1), we simplified the construction process by enabling the user to select the starting node type as being a disease, gene or drug. Once the starting node type has been selected, the user can choose to add additional features to the network, such as genetic data, interactions among proteins, the drugs that target them and the side effects associated with those drugs. Different types of networks (e.g. disease, gene or drug) offer complementary views from different perspectives. Next, a case study is presented starting with the network from disease nodes as an example.

### Global Disease gene network

**Figure 2.**
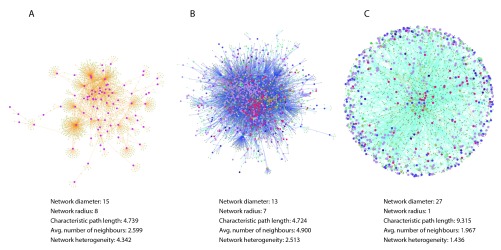
Human disease-gene networks. Networks were generated using iCTNet 2.0 for diseases represented in the GWAS Catalog ( **A**), the Comparative Toxicogenomics Database ( **B**) and OMIM ( **C**). Note the different topological characteristics (described below each network), particularly between **A** and **C**. Topological analysis was performed with Network analysis (a Cytoscape Core app).

Starting from any phenotype(s) in the database, users can add gene, drug and tissue directly (if connections among them exist), and secondly add miRNA, side effects and PPIs to further grow the network. As an example, we created three disease (phenotype)-gene networks by selecting all data available in the GWAS Catalogue (threshold p-value 1E ^-7^), CTD and OMIM databases. The connected component of each network was markedly different in size and topological properties. The GWAS network was comprised of 1547 nodes (82 diseases + 1465 genes) connected through 2010 edges (ratio N/E = 0.77), the CTD network included 5166 nodes (1168 diseases + 3998 genes) and 12657 edges (N/E = 0.41) and the OMIM network was formed by 2265 nodes (699 diseases + 1566 genes) and 2228 edges (N/E = 1.01). Upon layout within Cytoscape (spring embedded) a clearly distinct topology emerged for each network, with the GWAS network displaying a wheel and spoke pattern with most diseases at the center ( Figure 2A), and the OMIM network displaying a circular symmetric pattern, with most diseases towards the periphery ( Figure 2C). The CTD network displayed a pattern that resembled an aggregate of the other two, an expected outcome given that this database includes information on both common and rare diseases ( Figure 2B). The different topology between GWAS and OMIM networks clearly reflects the type of information each database contains. The central disposition of most diseases in the GWAS network (and the larger proportion of genes to diseases) highlights their polygenic nature and reflects the large amount of gene (locus) sharing among common diseases, consistent with our current understanding of their pathogenesis. On the other hand, the peripheral disposition of diseases in the OMIM network is a reflection of the limited genetic sharing characteristic of monogenic diseases, which dominate this database. Consistent with these observations, a network analysis conducted within Cytoscape showed differences between GWAS and OMIM networks in several parameters, including centrality, neighborhood connectivity and shortest path length distributions ( Figure 2). By using the “create similarity network” feature (located in the App menu) a user can convert disease-gene networks into disease-disease similarity networks (i.e. from bi-partite to homogeneous), in which two diseases are connected if a user-specified threshold of shared genes is met.

### The autoimmune disease set (autoimmunome)

**Figure 3.**
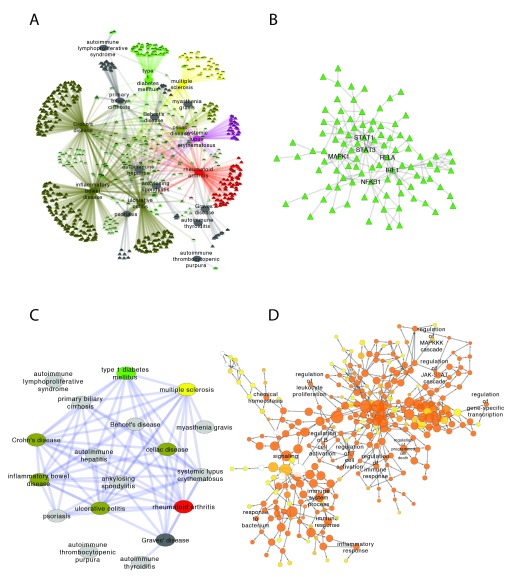
The autoimmune disease network. ( **A**) Common autoimmune diseases and their associated genes (according to the GWAS catalog) are displayed. ( **B**) Genes associated with multiple autoimmune diseases form a densely connected network at the protein level. ( **C**) Disease similarity network created from ( **A**). Two diseases are connected with more than 2 genes are shared. ( **D**) Gene ontology analysis of the genes in ( **B**) shows over-representation of immune related proteins.

We next downloaded the GWAS disease-gene network for 18 common autoimmune diseases (and their first degree protein interactions) ( Figure 3A). A clear pattern of gene sharing can be observed (green triangles in the center of the network represent shared genes between at least two diseases), consistent with our understanding on the genetic commonalities among autoimmune diseases. Using standard Cytoscape procedures (i.e. selected node type = genes and then created a new network), we further filtered this network to obtain only the protein interactome associated with more than one autoimmune disease ( Figure 3B). A highly connected component (n=98) emerged (N/E = 0.60) with several key genes of known immunological function (e.g. STAT1, STAT3, NFKB1, RELA and MAPK1) at its center. Using the “create similarity network” feature, diseases with more than 2 shared genes were connected in a new graph ( Figure 3C). To further explore the biological relevance of these nodes, a gene ontology analysis was performed on this network using the BiNGO App ^22^ and results were displayed as a new network ( Figure 3D). Confirming our previous observations, the set of genes associated with multiple autoimmune diseases is highly enriched (as indicated by the orange colored nodes) in immunological processes ranging from levels as general as leukocyte proliferation, and regulation of immune response, to as specific as regulation of MAPKKK and JAK-STAT cascades.

### Drug indications for autoimmune diseases

**Figure 4.**
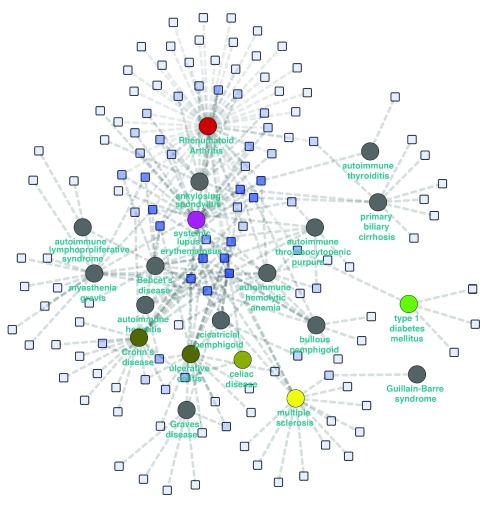
Autoimmune disease-drug indications network. Increased sharing of indications can be readily detected among diseases of similar etiology. Drugs are represented by blue squares, and the opacity of the square is proportional to its degree, thus shared drugs appear darker. Diseases are represented as circles.

In an attempt to evaluate the current pharmacological landscape in autoimmune disease treatment, we added all drugs known to be used to treat each autoimmune disease in the network according to CTD. As observed for genetic associations, while most treatments are disease-specific, there is substantial sharing of treatment modalities among multiple diseases ( Figure 4). This suggests that drug repurposing is a plausible strategy for diseases with shared genetic susceptibility and pathophysiological mechanisms.

## Conclusion

The iCTNet2 database and Cytoscape app are a systematically-developed resource and tool for studies requiring integration of multi-domain biological information. iCTNet2 illustrates how powerful the integration of heterogeneous biological interactions can be, through a simple and user-friendly interface. Comprehensive views of a given disease, including its genetic risk, gene expression profile, biological pathways affected, and actual and potential therapeutic options are just a few clicks away. Similarly, global landscapes of entire groups of diseases (i.e. malignancies, autoimmune disorders, etc) and their relevant “data neighbourhoods” can be easily created. Being a Cytoscape app, iCTNet2 also provides flexibility to conduct further analysis on the generated networks for further exploration, such as disease gene prediction, module detection, and topological network analysis.

## Software availability

### Software access

The App is available via the Cytoscape App Store.

### Latest source code

The source code can be accessed at https://github.com/LiliWangQueensu/iCTNet2_v2

### Archived source code as at the time of publication

https://zenodo.org/record/21386#.VbIoo_JzbIU

DOI 10.5281/zenodo.21386

### Software license

MIT license

## Data Availability

**Supplementary Data S1.** Mapping file between Genomics Institute of the Novartis Research Foundation (GNF) and Brenda Tissue Ontology (BTO). Click here for additional data file.. Click here for additional data file. **Supplementary Data S2.** Mapping file between Brenda Tissue Ontology (BTO) and the Human disease ontology (DO). Click here for additional data file.. Click here for additional data file. **Supplementary Data S3.** Mapping file between Brenda Tissue Ontology (BTO) and the Medical Dictionary for Regulatory Activities (MedRA). Click here for additional data file..
