# Supplementary material for: iCTNet2: integrating heterogeneous biological interactions to understand complex traits
Source: F1000Res. 2015 Sep 28;4:485. Originally published 2015 Aug 5. [Version 2] doi: 10.12688/f1000research.6836.2 (PMC4706053; doi:10.12688/f1000research.6836.2)
Supplement: Peer review history

# Peer review: Referee response for version 2

**DOI:** 10.5256/f1000research.7667.r10539

**Published:** 2015-12-29

**Authors:** Gary Bader

**Affiliations:** 1 Department of Computer Science, University of Toronto, Toronto, ON, Canada

The authors have addressed all the concerns raised in the last review.  The manuscript reads much more smoothly and clearly now.

I have read this submission. I believe that I have an appropriate level of expertise to confirm that it is of an acceptable scientific standard.

# Peer review: Referee response for version 1

**DOI:** 10.5256/f1000research.7350.r9868

**Published:** 2015-09-02

**Authors:** Gary Bader

**Affiliations:** 1 Department of Computer Science, University of Toronto, Toronto, ON, Canada

The authors describe a Cytoscape app that provides the entry point to the iCTNet resource, containing networks connecting a variety of concepts, including genes, drugs, side effects, tissues, miRNAs and phenotypes. This resource is very useful for users wishing to start with one of these information types and navigate to others e.g. to find all genes and drugs involved in a disease of interest. In general the app works very smoothly. My comments relate mainly to missing details and text to be clarified, as detailed below.

Major points:

P5 “Next, a case study is presented starting with the network from disease nodes as an example.” It would be useful to describe the full workflow, including the scientific question, rationale and end goal.

There is a “Create similarity network” feature present in the App menu, but this is not described in the manuscript. It would be useful to add a section describing the feature and a use case.

If the user loads too much data, the app will take a long time to respond and the process can’t easily be canceled. The user should be warned in the manuscript or via the app that large queries may take a long time.

Search starting points can be gene, disease or drug. Why can’t users search by other starting points e.g. tissue?

The last update date of the ICT database and the date and version used for each resource should be clearly communicated to the user e.g. via the manuscript, app and/or ICT website.

Minor points:

Page 2: clarify “stacked onto”.

P2: “ids” -> identifiers

P3: “The CTD (12)” – what does ‘(12)’ mean?

P3 – drugs paragraph. This section is a bit unclear. CTD provides references to drugbank identifiers? Should it be that CTD provides references to drugbank records?  How is the ‘function’ of drugs defined – is this just the drug target?  Drugbank contains fewer entries compared to what?

P3 – “phenotype-gene” section.  “To convert from SNP to gene associations, we combined overlapping loci for each GWAS Catalog disease17.”  How were the loci combined?

“The mode author reported gene for each loci was selected as primary.” – what is a mode?

Page 3 and 4 – in the “Types of interactions” section, all sub-sections should include the number of interactions e.g. how many gene-tissue interactions are there?

P5 – what is a metagraph?

P5 – “spike” -> “spoke”?

P5 – “Using standard Cytoscape procedures, we further filtered this network to obtain only the protein interactome associated with more than one autoimmune disease (Figure 3B)” – the Cytoscape procedures should be detailed to make it easier for users to replicate the results in the manuscript.

Figure 1 – the tissues circle of nodes is covered by edges from other circles – can it be moved out a bit to show how it connects to other circles?

I have read this submission. I believe that I have an appropriate level of expertise to confirm that it is of an acceptable scientific standard, however I have significant reservations, as outlined above.

# Response

**Published:** 2015-09-19

**Authors:** Sergio Baranzini

**Affiliations:** University of California San Francisco, USA

Thank you for your comments!

Please see a point-by point answer below

**Major points:**

**P5 “Next, a case study is presented starting with the network from disease nodes as an example.” It would be useful to describe the full workflow, including the scientific question, rationale and end goal.**

This has been added to the revised manuscript

**There is a “Create similarity network” feature present in the App menu, but this is not described in the manuscript. It would be useful to add a section describing the feature and a use case.**

We have expanded the manuscript to describe this feature in detail and have modified Figure 3 to include an example of this feature. 

If the user loads too much data, the app will take a long time to respond and the process can’t easily be canceled. The user should be warned in the manuscript or via the app that large queries may take a long time.

We have introduced a warning message for large networks. Specifically, the message will be shown if: 

(1) the size of query diseases > 50 and PPI depth>0; or the size of 

query diseases > 100;

(2) the size of query genes >500 and PPI depth >0; or the size of query 

genes > 1000;

(3) the size of query drugs > 100 and PPI depth >0; or the size of query 

drugs > 200;

**Search starting points can be gene, disease or drug. Why can’t users search by other starting points e.g. tissue?**

Technically, this should be possible. however, with the three provided starting points, all other searches are technically possible using basic Cytoscape functions. 

**The last update date of the ICT database and the date and version used for each resource should be clearly communicated to the user e.g. via the manuscript, app and/or ICT website.**

Table 1 has been updated.

**Minor points:**

**Page 2: clarify “stacked onto”.**

done

**P2: “ids” -> identifiers**

done

**P3: “The CTD (12)” – what does ‘(12)’ mean?**

removed (12)

**P3 – drugs paragraph. This section is a bit unclear. CTD provides references to drugbank identifiers? Should it be that CTD provides references to drugbank records?  How is the ‘function’ of drugs defined – is this just the drug target?  Drugbank contains fewer entries compared to what?**

This paragraph has been re-written

**P3 – “phenotype-gene” section.  “To convert from SNP to gene associations, we combined overlapping loci for each GWAS Catalog disease17.”  How were the loci combined?**

We have provided a reference detailing how this was done. Basically, we proceeded as follows:

Lead-SNPs were assigned windows—regions wherein the causal SNPs are assumed to lie—retrieved from the DAPPLE server. Windows were calculated for each lead-SNP by finding the furthest upstream and downstream SNPs where *r*2 > 0.5 and extending outwards to the next recombination hotspot. Associations were ordered by confidence, sorting on following criteria: high/low confidence, p-value (low to high), and recency. In order of confidence, associations were overlapped by their windows into disease-specific loci. By organizing associations into loci, associations from multiple studies tagging the same underlying signal were condensed.

**“The mode author reported gene for each loci was selected as primary.” – what is a mode?**

Corrected

**Page 3 and 4 – in the “Types of interactions” section, all sub-sections should include the number of interactions e.g. how many gene-tissue interactions are there?**

The numbers of interactions are now specified.

**P5 – what is a metagraph?**

We refer to a metagraph as the graph describing the interactions among the different node types.

**P5 – “spike” -> “spoke”?**

done

**P5 – “Using standard Cytoscape procedures, we further filtered this network to obtain only the protein interactome associated with more than one autoimmune disease (Figure 3B)” – the Cytoscape procedures should be detailed to make it easier for users to replicate the results in the manuscript.**

Done

**Figure 1 – the tissues circle of nodes is covered by edges from other circles – can it be moved out a bit to show how it connects to other circles?**

Done

# Peer review: Referee response for version 1

**DOI:** 10.5256/f1000research.7350.r9869

**Published:** 2015-08-24

**Authors:** Amitabh Sharma

**Affiliations:** 1 Department of Medicine, Harvard Medical Center, Boston, MA, USA

Baranzini *et al. *updated the iCTNet database from the data collected from public repositories to facilitate the building, visualization and analysis of heterogeneous biological networks in a comprehensive fashion via the Cytoscape platform. I like the manuscript and source developed by Baranzini group. The resource update is important and timely, and is well-done and clearly described. It is freely available and provides a good resource for the community to understand the connections between different omics or the big data in disease medicine.

A few minor comments would improve the manuscript:

- Add some text in the conclusion about how version 2 is better than version 1.
- The Phentoypes/diseases vocabulary sources do not overlap much, did this result in a lot of data loss while integrating?
- Add some description regarding the edges in the Figure 2 legend. What are different node colors? GWAS network is much sparse because of the incompleteness of the interactome and also we have literature bias for the OMIM data.
- In figure 3, is the network PPI only or aggregated network of all sources? Also, Figure 3C should include only those terms that are below specific thresholds, like p<0.05.

Overall, an excellent work.

I have read this submission. I believe that I have an appropriate level of expertise to confirm that it is of an acceptable scientific standard.
